# Epidemiological and Laryngoscopic Characteristics of Head and Neck Burns Requiring Airway Evaluation: A Single-Center Retrospective Cohort

**DOI:** 10.3390/medicina62101837

**Published:** 2026-09-23

**Authors:** Mehmet Necmeddin Sutaşır, Mustafa Turan, Mohamed Mukhtar Mohamed, Mehmet Tatlı, Murat Özmen, Gönül Karataş, Nihat Müjdat Hökenek

**Affiliations:** 1 Department of Emergency Medicine, Başakşehir Çam and Sakura City Hospital, University of Health Sciences, Istanbul 34480, Turkeynihathokenek@gmail.com (N.M.H.); 2 Department of Burn Center and Burn Intensive Care Unit, Başakşehir Çam and Sakura City Hospital, University of Health Sciences, Istanbul 34480, Turkey; 3 Department of Emergency Medicine, Mogadishu Somali Turkiye Training and Research Hospital, Faculty of Medicine & Surgery, Salaam University, Mogadishu BN 00252, Somalia; drmohamuqtar@gmail.com; 4 Department of Emergency Medicine, Van Training and Research Hospital, University of Health Sciences, Van 65100, Turkey

**Keywords:** head and neck burns, laryngoscopy, inhalation injury, airway management, burn etiology, epidemiology

## Abstract

*Background and Objectives*: Head and neck burns are a clinically demanding subset of burn injury because of their potential for life-threatening airway compromise, cosmetic disfigurement, and psychological morbidity. Although systematic flexible fiberoptic laryngoscopy has been advocated for airway triage in this population, real-world epidemiological and laryngoscopic data from mixed pediatric and adult cohorts, particularly outside high-income Western settings, remain limited. This study aimed to describe the epidemiological characteristics, anatomical distribution, laryngoscopic findings, and airway management outcomes of patients with head and neck burns who underwent flexible fiberoptic laryngoscopy for a clinical indication of suspected airway involvement at a Turkish tertiary burn center. Because inclusion required a laryngoscopy indication, patients with low-risk, isolated facial burns without airway concern were not represented in this cohort, and the reported proportions should not be interpreted as population-level prevalence estimates. *Materials and Methods:* This retrospective, single-center observational study analyzed 279 patients with head and neck burns who underwent flexible fiberoptic laryngoscopy at a tertiary burn center between June 2022 and December 2024. Burn etiology was undocumented in 117 patients (41.9%); inferential comparison of etiology between age groups was therefore restricted to the 162 patients with documented etiology, using the chi-square test. Total body surface area (TBSA) and burn depth were not systematically recorded in the electronic health record during the study period and could not be analyzed. *Results:* Mean age was 25.4 ± 22.6 years (43.4% pediatric, 56.6% adult). Among the 162 patients (58.1%) with documented etiology, scalding predominated in children (57.4%), whereas flame (31.9%) and explosion injuries (23.4%) were more common in adults (χ^2^ = 68.23, df = 4, *p* < 0.001); as etiology was undocumented in the remaining 41.9% of the cohort, this comparison should be interpreted with caution. The face (70.3%) and upper airway (42.3%) were the most frequently involved regions. Laryngoscopy showed mucosal edema in 41.2% and hyperemia in 19.4% of patients. Endotracheal intubation was required in 4.7% and tracheostomy in 1.8% of patients; no mortality attributable to head and neck burns was observed. *Conclusions:* Because etiology was undocumented in 41.9% of the cohort, the observed age-related differences in documented burn etiology should be regarded as hypothesis-generating rather than definitive, despite reaching statistical significance. Similarly, because standardized burn-severity metrics were unavailable, the low airway-intervention rate cannot be causally attributed to the laryngoscopic protocol and more plausibly reflects the heterogeneous, predominantly mild-to-moderate severity spectrum of the cohort. These findings are best regarded as descriptive and hypothesis-generating, underscoring the need for prospective, multicenter studies with standardized severity documentation to support evidence-based airway management protocols in head and neck burn patients.

## 1. Introduction

Burn injuries constitute a substantial global health burden, accounting for an estimated 180,000 deaths annually and millions of non-fatal injuries requiring medical attention [1,2]. Head and neck burns represent a particularly demanding subset, given their potential to compromise the airway and to cause severe cosmetic disfigurement and lasting psychological morbidity [3,4]. The anatomical complexity of this region—encompassing the face, ears, scalp, neck, and upper respiratory tract—requires a systematic approach to evaluation and management.

Inhalation injury, frequently associated with facial burns, remains one of the leading causes of mortality among burn patients [5,6]. The upper airway is susceptible to thermal injury from heated gases, which can produce rapid mucosal edema and life-threatening airway obstruction [7]. Early recognition of airway involvement through clinical assessment and laryngoscopic evaluation is therefore crucial for timely intervention [8,9]. Flexible fiberoptic laryngoscopy has emerged as a valuable tool for evaluating upper airway burns, allowing direct visualization of supraglottic and glottic structures [10,11].

The epidemiology of burn injury varies considerably across populations and age groups [12,13]. Pediatric burns, which account for a substantial proportion of all burn admissions, are predominantly caused by scalding, particularly in children under five years of age [14,15,16]. Adult burns, in contrast, more frequently result from flame, electrical, and occupational exposures [17,18]. Understanding these age-specific patterns is essential for developing targeted prevention strategies and optimizing clinical management.

Despite advances in burn care, data on the specific characteristics of head and neck burns in populations spanning both pediatric and adult age groups remain limited [19]. Most existing studies address either general burn epidemiology or a single subpopulation, and few provide detailed analysis of anatomical distribution together with laryngoscopic findings [20,21]. There is, moreover, a relative paucity of literature from developing and rapidly urbanizing regions, where burn injury patterns may differ from those reported in Western populations.

The aim of this study was to describe the epidemiological characteristics, anatomical distribution, laryngoscopic findings, and airway management outcomes of patients with head and neck burns at a Turkish tertiary burn center, in a cohort spanning both pediatric and adult age groups. Given the scarcity of real-world observational data from rapidly urbanizing countries and the value of externally validating established airway management strategies across diverse clinical settings, the study was designed as a descriptive, hypothesis-generating investigation rather than a confirmatory efficacy trial.

## 2. Materials and Methods

### 2.1. Study Design and Setting

This retrospective, single-center observational study was conducted at Başakşehir Çam and Sakura City Hospital, a tertiary referral center with a dedicated burn unit and burn intensive care unit, in Istanbul, Turkey. The study period extended from June 2022 to December 2024. Ethical approval was obtained from the Health Sciences University Başakşehir Çam and Sakura City Hospital Clinical Research Ethics Committee (Decision No: 318; Date: 22 October 2025). Because of the retrospective, observational design and the use of fully anonymized data, the requirement for individual informed consent was waived. The study was conducted in accordance with the Declaration of Helsinki and is reported following the STROBE guideline for observational studies. As inclusion required that flexible fiberoptic laryngoscopy be performed for a clinical airway indication, this cohort represents a laryngoscopy-selected sample of head and neck burn patients rather than an unselected epidemiological population.

### 2.2. Patient Selection

Patients were identified through systematic review of the hospital’s electronic medical records. Inclusion criteria were (1) documented burn injury involving the head and neck region (face, ears, neck, scalp, or upper respiratory tract); (2) laryngoscopic airway evaluation performed because of suspected inhalation injury, facial or upper airway burns, or a clinical risk indicator (hoarseness, stridor, dyspnea, oropharyngeal soot, or singed nasal hairs); and (3) patients of any age managed in the burn unit, burn intensive care unit, or burn outpatient clinic. Laryngoscopic evaluation was triggered whenever at least one of these risk indicators, or an equivalent clinical concern, was documented by the treating burn or emergency medicine team. These indications were applied consistently as institutional practice throughout the study period, but adherence was not formally audited at the level of the individual physician; residual variation in clinical threshold therefore cannot be excluded (see Section 5).

Exclusion criteria were (1) patients intubated before arrival at the study facility; (2) concomitant major head trauma; (3) pre-existing laryngeal or tracheal pathology; (4) more than 30% of critical data missing from the medical record; (5) technically inadequate laryngoscopy images precluding evaluation; and (6) patients who left against medical advice.

Because inclusion required that laryngoscopy be performed, the cohort represents the subset of head and neck burn patients selected for airway risk assessment rather than the full spectrum of head and neck burns presenting to the center; patients whose burns did not involve the face or upper airway and who lacked any risk indicator for inhalation injury would not have met the inclusion criteria and are therefore not represented. The reported anatomical distribution and airway intervention rates should be interpreted within this selected population. Exclusion Criterion 4 (>30% of critical data missing) was applied to core eligibility and characterization variables (age, sex, anatomical site, laryngoscopic findings, and intervention status). Etiology and burn severity metrics (TBSA, burn depth) were not classified as “critical data” under this criterion, as these fields were recognized to be systematically, rather than randomly, absent from the source database (see Section 2.3).

### 2.3. Data Collection

Data were extracted from the hospital’s electronic health record system and included demographic information (age, sex), burn etiology (scalding, flame, electrical, chemical, explosion, contact), anatomical distribution of burns, date and season of presentation, and clinical outcomes. Burn depth and total body surface area (TBSA) were not systematically documented in the electronic medical record during the study period and could therefore not be included as study variables; this omission is a fundamental limitation with direct consequences for the interpretation of the airway management findings, discussed in detail in Section 5. Surrogate indicators of injury severity, such as intensive care unit admission, hospital length of stay, need for mechanical ventilation, or operative burn treatment, were likewise not captured in a structured, analyzable format and could not be incorporated as study variables.

All patients underwent flexible fiberoptic laryngoscopy performed by otolaryngology specialists. Laryngoscopy was performed using a standard-caliber flexible fiberoptic scope via the transnasal route, under topical nasal anesthetic/decongestant spray. Procedural sedation was administered per institutional protocol in young children when required, without sedation in cooperative patients. All examinations were performed or directly supervised by an attending otolaryngologist. Findings were assessed live at the bedside. Operational definitions were as follows: mucosal edema was defined as visible mucosal swelling causing partial airway lumen narrowing; hyperemia/erythema was defined as diffuse mucosal redness without associated swelling or exudate; and excessive secretion was defined as pooling of mucus, saliva, or soot-stained secretions obscuring clear visualization of the airway lumen. Structures systematically evaluated included the nasopharynx, oropharynx, hypopharynx, larynx, epiglottis, vocal cords, and arytenoid regions. Documented findings included mucosal edema, hyperemia/erythema, excessive upper airway secretions, airway patency, and vocal cord mobility. The interval between burn injury and laryngoscopic evaluation was not consistently captured as a structured, analyzable field. It could not be reliably reconstructed for the full cohort, so time-dependent changes in airway findings during the first 24–48 h after injury could not be analyzed. Repeat laryngoscopy in patients with mucosal edema on initial evaluation was performed at the treating team’s discretion when clinical status evolved, rather than on a fixed follow-up schedule, and the frequency of repeat examinations was not systematically recorded. Likewise, whether a given airway intervention was performed immediately after laryngoscopy or after subsequent clinical deterioration was not consistently coded as a discrete variable and could not be systematically reported.

Otoscopic examination was performed in patients with auricular involvement and anterior rhinoscopy in those with suspected nasal involvement; because these examinations were performed only when clinically indicated rather than as part of a fixed protocol, their findings are not representative of the full cohort and are reported accordingly (Section 2.5, Table 1). Treatment recommendations, hospitalization status, and clinical outcomes were recorded.

For anatomical localization, burns involving multiple sites were recorded independently for each anatomical region; because patients could have burns affecting more than one anatomical area simultaneously, percentages for different regions are not mutually exclusive.

### 2.4. Statistical Analysis

Statistical analyses were performed using SPSS version 26.0 (IBM Corp., Armonk, NY, USA). Continuous variables were assessed for normality using the Shapiro–Wilk test. Normally distributed continuous variables are presented as mean ± standard deviation (SD); non-normally distributed variables are presented as median (interquartile range, IQR). Categorical variables are expressed as frequencies and percentages. Between-group comparisons used the chi-square test or Fisher’s exact test for categorical variables and the independent-samples *t*-test or Mann–Whitney U test for continuous variables, as appropriate. A *p*-value < 0.05 was considered statistically significant.

For the inferential comparison of burn etiology between pediatric and adult patients, cases with undocumented etiology (n = 117, 41.9%) were excluded. Etiological categories with small expected cell frequencies (chemical, contact, and hot-oil burns) were combined into a composite “Other” category to satisfy chi-square assumptions (expected frequency ≥ 5 per cell). All etiological percentages are reported with explicitly specified denominators: proportions based on the full cohort (n = 279) for descriptive context and proportions among analyzable cases (n = 162) for inferential comparison. Because the original database was structured for descriptive epidemiological reporting rather than for comparative analysis of missing-data patterns, the variables required to formally compare patients with documented versus undocumented etiology were not retained in an analyzable format after anonymization; this comparison could therefore not be performed retrospectively (see Section 5).

Multivariable logistic regression to identify independent predictors of airway intervention was not performed because the number of intervention events (n = 18) fell below the widely applied threshold of 10 events per candidate variable required for stable multivariable modeling. All analyses are therefore descriptive and exploratory, and no clinical prediction rule should be derived from the present findings.

Diagnostic performance of mucosal edema and stridor for predicting definitive airway intervention was assessed using sensitivity, specificity, positive predictive value (PPV), and negative predictive value (NPV), with 95% confidence intervals calculated using the Wilson score method.

### 2.5. Reporting of Selective-Examination Findings

Because otoscopic and rhinologic examinations were performed only in patients with a clinical indication, and because the number of patients undergoing each selective examination was not systematically recorded, the denominators for these findings (Table 1) are reported against the full cohort (n = 279) for contextual reference only; the true prevalence within each examined subgroup cannot be estimated from the present data and should not be interpreted as population-level prevalence.

**Table 1:** Physical examination findings and clinical outcomes (*n* = 279).

Variable	n	%
Laryngoscopic findings (n = 279)		
Mucosal edema present	115	41.2
Hyperemia/erythema present	54	19.4
Excessive upper airway secretions present	42	15.1
Larynx assessed as normal	72	25.8
Vocal cords normal/mobile	124	44.4
Epiglottis normal	56	20.1
Airway patent (explicitly documented)	62	22.2
Stridor present	7	2.5
Otologic findings †		
Auricular burn	39	14.0 ‡
Tympanic membrane normal	32	11.5 ‡
Otorrhea	19	6.8 ‡
Cerumen impaction	12	4.3 ‡
Tympanic membrane perforation	5	1.8 ‡
Rhinologic findings †		
Nasal mucosal edema	37	13.3 ‡
Nasal cavity normal	3	1.1 ‡
Airway management (n = 279)		
Endotracheal intubation performed	13	4.7
Tracheostomy performed	5	1.8
Clinical outcome (n = 279)		
Close follow-up recommended	119	42.7
Additional hospitalization required	4	1.4
Mortality	0	0.0

† Percentages calculated against the full cohort (*n* = 279) for contextual reference. Otologic and rhinologic examinations were performed only in patients with a clinical indication; because the number examined in each subgroup was not systematically recorded, these figures should not be interpreted as population-level prevalence estimates. ‡ Assessed only among patients with a clinical indication for otoscopy (see Section 2.5); distinct from the general ear-burn rate (40.5%) reported in Section 3.3, which reflects external examination findings across the full cohort.

## 3. Results

### 3.1. Demographic Characteristics

A total of 279 patients meeting the inclusion criteria were enrolled during the 30-month study period. The study population spanned a broad clinical spectrum, ranging from minor facial burns to suspected severe inhalation injury. Mean age was 25.4 ± 22.6 years (median: 23.0 years; range: 0–83 years). The pediatric population (<18 years) comprised 121 patients (43.4%), and 158 patients (56.6%) were adults (≥18 years). Mean age was 3.8 ± 4.6 years in the pediatric group and 42.0 ± 15.8 years in the adult group. The most frequently affected age group was 18–40 years (27.6%), followed by 0–2 years (24.7%) and 41–65 years (23.3%) (Table 2).

Temporal distribution showed that 35 patients (12.5%) presented in 2022, 130 (46.6%) in 2023, and 114 (40.9%) in 2024. Seasonal distribution was relatively even: spring 28.0% (n = 78), autumn 27.2% (n = 76), summer 24.0% (n = 67), and winter 20.8% (n = 58).

### 3.2. Burn Etiology

In the full cohort (n = 279), burn etiology was not explicitly documented in 117 patients (41.9%), substantially constraining the interpretability of etiological analyses. Among the 162 patients with documented etiology (58.1% of the cohort), scalding (hot water, tea, or coffee) was the most frequently recorded mechanism (n = 42; 25.9% of analyzable cases, 15.1% of the full cohort), followed by electrical burns (n = 38; 23.5%/13.6%), flame burns (n = 35; 21.6%/12.5%), and explosion injuries (n = 25; 15.4%/9.0%). Chemical, contact, and hot-oil burns together comprised the remaining 13.6% of analyzable cases (n = 22) (Table 3).

Age-related differences in documented burn etiology were observed. Among pediatric patients with documented etiology (n = 68), scalding predominated (57.4%, n = 39), whereas flame burns were infrequent (7.4%, n = 5). Among adults with documented etiology (n = 94), flame burns were the most common mechanism (31.9%, n = 30), followed by explosion (23.4%, n = 22) and electrical injuries (23.4%, n = 22). After exclusion of cases with undocumented etiology, the distribution of burn mechanisms differed significantly between pediatric and adult patients (χ^2^ = 68.23, df = 4, *p* < 0.001). Etiology documentation was, however, missing in 41.9% of the cohort; therefore, a systematic bias cannot be excluded: if documentation failure is associated with injury severity, presenting mechanism, or mode of admission, the observed distribution may be directionally biased. As noted in Section 2.4, the anonymized analytical dataset did not retain the variables needed to formally test this possibility, so these etiological patterns should be read as descriptive and consistent with existing literature [15,22] rather than as definitive estimates.

### 3.3. Anatomical Distribution and Laryngoscopic Findings

Analysis of anatomical distribution showed that the face was the most commonly affected region, involved in 70.3% (n = 196) of patients. Upper airway involvement, defined as a laryngoscopic abnormality of the nasopharynx, oropharynx, hypopharynx, or larynx, was documented in 42.3% (n = 118). Ear burns, as documented on general physical examination across the full cohort, were present in 40.5% (n = 113); oral cavity involvement in 24.0% (n = 67), neck involvement in 13.3% (n = 37), and scalp involvement in 1.4% (n = 4). Because patients frequently had burns affecting more than one anatomical site, these percentages are not mutually exclusive (Table 1).

Laryngoscopic evaluation was performed in all 279 patients, each of whom met the predefined clinical indications for airway assessment. Mucosal edema was identified in 115 patients (41.2%), and hyperemia/erythema in 54 patients (19.4%). Excessive upper airway secretions were documented in 42 patients (15.1%). The larynx was assessed as normal in 72 patients (25.8%), bilateral vocal cord mobility was preserved in 124 patients (44.4%), and the epiglottis appeared normal in 56 patients (20.1%). Explicit documentation of a patent airway was available in 22.2% of cases. Stridor was present in 7 patients (2.5%).

Otoscopic and rhinologic examinations were performed only in patients with a clinical indication; as noted in Section 2.5, their findings are therefore not representative of the full cohort (n = 279) and are presented accordingly. These otoscopy-confirmed findings should be distinguished from the general ear-burn figure reported above (Section 3.3, 40.5%), which reflects external burns documented on physical examination across the full cohort rather than otoscopically confirmed auricular involvement. Among patients who underwent otoscopic examination, auricular burns were documented in 39 patients (14.0% of the full cohort), normal bilateral tympanic membranes in 32 patients (11.5%), otorrhea in 19 patients (6.8%), cerumen impaction in 12 patients (4.3%), and tympanic membrane perforation in 5 patients (1.8%). Among patients who underwent rhinologic assessment, nasal mucosal edema was identified in 37 patients (13.3%) and a normal nasal cavity in 3 patients (1.1%) (Table 1).

### 3.4. Characteristics of Patients Requiring Airway Intervention

Endotracheal intubation was performed in 13 patients (4.7%) and tracheostomy in 5 patients (1.8%), for a total of 18 patients (6.5%) requiring definitive airway intervention. Table S1 summarizes the aggregate characteristics of this subgroup. Among these 18 patients, the majority were adults (n = 14, 77.8%), and flame or explosion burns were the documented etiology in 12 cases (66.7%). Laryngoscopic findings included mucosal edema in all 18 patients (100%), with stridor documented in 7 of 18 (38.9%). All 18 patients had concurrent upper airway involvement on laryngoscopy, and facial burns were present in 15 of 18 (83.3%). Burn severity data (TBSA, depth) were unavailable for this subgroup, as for the remainder of the cohort. Because this retrospective study was based on anonymized aggregate data extracted from the institutional database, the individual patient-level variables required for a case-by-case supplementary table were not available for secondary extraction; the airway-intervention subgroup is therefore presented in aggregate form (Table S1).

Diagnostic performance analysis showed that mucosal edema had 100.0% sensitivity (95% CI 82.4–100.0) and 100.0% NPV (95% CI 97.7–100.0) for definitive airway intervention but modest specificity (62.8%, 95% CI 56.8–68.5) and PPV (15.7%, 95% CI 10.1–23.4). In contrast, stridor, although present in only 7 of the 18 intervention patients (38.9%, 95% CI 20.3–61.4), was present exclusively among patients who required intervention, yielding 100.0% specificity and PPV (95% CI 98.5–100.0 and 64.6–100.0, respectively) and 96.0% NPV (95% CI 92.9–97.7) (Table S2).

### 3.5. Treatment and Clinical Outcomes

For patients who did not require definitive airway intervention, supportive medical therapy was implemented according to laryngoscopic findings, including humidified oxygen therapy, nebulized racemic epinephrine for mucosal edema, and systemic corticosteroids in selected patients with significant airway inflammation; these interventions reflect local clinical practice rather than a standardized, evidence-based protocol validated in this population. Close clinical follow-up was recommended for 119 patients (42.7%). Topical treatments included ear drops in 13 patients (4.7%) and local wound dressings in 12 patients (4.3%). Imaging studies were obtained in 6 patients (2.2%).

Most patients (98.6%, n = 275) were managed according to the existing treatment plan, with only 4 patients (1.4%) requiring additional hospitalization. No mortality attributable to head and neck burns was observed during the study period. As discussed in Limitations, this finding should be interpreted in light of the unavailability of standardized burn-severity data for this cohort.

## 4. Discussion

This study provides descriptive data on the epidemiological characteristics, anatomical distribution, and clinical outcomes of head and neck burns evaluated at a Turkish tertiary burn center. It documents age-related patterns in burn etiology and the spectrum of laryngoscopic findings in a mixed pediatric and adult population, within the methodological limits discussed below.

The bimodal age distribution, with peaks in early childhood (0–2 years) and young adulthood (18–40 years), matches patterns reported in the global burn epidemiology literature [1,2]. The high proportion of pediatric patients (43.4%) reflects the known vulnerability of children under five years of age to burn injury, consistent with systematic reviews identifying this group as bearing the highest burn risk, predominantly from scalds [14,23].

Etiology was undocumented in 41.9% of the full cohort; with this caveat in mind, age-related differences in documented etiology were observed among the 162 patients with documented data: scalding was far more common in children (57.4%) than in adults (3.2%), while flame and explosion burns predominated in adults (31.9% and 23.4%, respectively; χ^2^ = 68.23, df = 4, *p* < 0.001) [15,22]. Clinically, the predominance of scalding in young children supports targeted education on safe bathing practices and secure storage of hot liquids, since most pediatric scald burns are considered preventable [24]. For adults, the etiological pattern points toward workplace safety programs addressing flame and electrical hazards [17,18].

The face (70.3%) was the most frequently affected region, followed by the upper airway (42.3%) and ears (40.5%). This reflects the exposed nature of the head and neck region and supports current guidance to prioritize airway evaluation in patients with facial burns [7,8]. Systematic laryngoscopy appeared most informative in patients with facial burns, stridor, or another recognized risk indicator: all 18 patients who ultimately required airway intervention had laryngoscopic evidence of upper airway involvement, and 83.3% had facial burns. Patients without these features may derive less incremental benefit from routine laryngoscopy, though this cannot be confirmed without a comparator group.

Mucosal edema (41.2%) and hyperemia (19.4%) were the most common laryngoscopic findings, consistent with published data supporting flexible fiberoptic laryngoscopy as a reliable tool for assessing upper airway injury, including its high predictive value for the need for intubation [10,11].

Our intubation (4.7%) and tracheostomy (1.8%) rates were lower than in series focused specifically on confirmed inhalation injury but consistent with studies spanning a broader clinical spectrum of head and neck burns. These findings parallel the results of Moshrefi et al., who found that flexible fiberoptic laryngoscopy allowed 98% of burn patients meeting traditional intubation criteria to be safely monitored without intervention [11]; historically, airway decisions relied heavily on clinical suspicion, and many intubations performed before transfer to a burn center have since been judged unnecessary [25].

Zero mortality is reassuring but, as noted above, most likely reflects cohort severity rather than protocol efficacy, since series focused on confirmed inhalation injury report substantially higher mortality [5,6]. Inhalation injury is known to increase mortality risk and to affect morbidity for years after injury [1]. Seasonal distribution was relatively even, with a slight spring and autumn predominance; this differs from reports of winter peaks linked to heating, and regional differences in climate and heating practices likely explain the discrepancy [22,26].

These diagnostic performance characteristics suggest a complementary, two-tiered role for these findings in clinical triage: the near-perfect NPV of mucosal edema supports its use as a screening finding, whereas its modest specificity means edema alone should not trigger intervention. Conversely, the perfect specificity and PPV of stridor suggest it may serve as a highly reliable trigger for urgent airway intervention when present, despite its low sensitivity. These observations should be validated prospectively before informing clinical decision-making.

## 5. Limitations

First, the retrospective, single-center design limits the generalizability of the findings to other populations and healthcare settings. Because inclusion required that laryngoscopy be performed, the cohort represents the subset of head and neck burn patients selected for airway risk assessment rather than the full spectrum of head and neck burns seen at the center (Section 2.2).

Second, and most critically, burn depth and TBSA were not systematically documented during the study period and could not be included as study variables. Without burn-severity data, the study cannot distinguish between two fundamentally different explanations for the low intubation rate: (i) that the laryngoscopic protocol enabled safe avoidance of unnecessary intubation, or (ii) that most patients simply did not require intubation, irrespective of the assessment strategy, because of predominantly minor-to-moderate burn injury. The low intervention rates and zero mortality observed in our cohort are therefore more appropriately attributed to this heterogeneous, predominantly mild-to-moderate severity spectrum than to the effectiveness of the laryngoscopic protocol. The absence of severity data also precluded surrogate-severity analyses (ICU admission, length of stay, mechanical ventilation, operative treatment) and multivariable regression to identify independent predictors of airway intervention. Future prospective studies should incorporate standardized severity metrics, including TBSA and burn-depth classification, to enable confounding-adjusted analyses and support validated airway management decision algorithms.

Third, the limited number of airway-intervention events (n = 18: 13 intubations and 5 tracheostomies) precluded formal multivariable logistic regression, since this event count falls below the widely applied threshold of 10 events per candidate variable; no clinical prediction rule should be derived from the present descriptive findings.

Fourth, burn etiology was undocumented in 41.9% of cases. A systematic bias cannot be excluded if documentation failure is related to injury mechanism, severity, or mode of presentation. Because the original database was constructed for descriptive epidemiological reporting rather than for comparative analysis of missing-data patterns, the variables needed to formally compare documented and undocumented cases were not retained in the anonymized analytical dataset, and this comparison could not be performed retrospectively. A sensitivity analysis assuming that all undocumented cases represent a single category would materially alter the reported proportions, so the reported etiological patterns should be read as indicative observations consistent with existing literature rather than as robust epidemiological estimates.

Fifth, otologic and rhinologic evaluations were performed only when clinically indicated rather than as part of a standardized protocol; asymptomatic patients were not examined. The reported prevalence of auricular burns, tympanic membrane perforation, nasal mucosal edema, and other regional findings therefore likely underestimates the true prevalence in the cohort and should be interpreted as indicative findings within the examined subgroup only, not as population-level prevalence estimates.

Sixth, several process-level variables relevant to airway management timing were not systematically captured in structured form, including the interval between burn injury and laryngoscopic evaluation, the frequency of repeat laryngoscopy in patients with mucosal edema, whether any patient required delayed intubation after an initially negative examination, and whether airway interventions were performed immediately after laryngoscopy or after subsequent clinical deterioration. These gaps limit the ability to characterize the time course of airway findings and management decisions and should be addressed through structured prospective data collection.

Diagnostic performance of mucosal edema and stridor was not stratified by age category, as the small number of intervention events (n = 18, further divided into 14 adults and 4 pediatric patients) would yield unstable estimates with very wide confidence intervals; age-stratified diagnostic accuracy should be examined in larger, prospective cohorts.

As this was a retrospective study without a systematically archived video record, inter-observer agreement for laryngoscopic findings could not be assessed; this should be addressed in future prospective studies.

Finally, the absence of long-term follow-up data precludes assessment of late complications, functional outcomes, and quality-of-life measures, which are important endpoints in head and neck burn care.

## 6. Conclusions

In this single-center, retrospective cohort of 279 patients with head and neck burns, burn mechanism was undocumented in 41.9% of the full cohort, and a systematic bias in the remaining, documented cases cannot be excluded (see Section 5). With this limitation explicitly noted, age-related differences in documented burn etiology were observed: scalding predominated in pediatric patients, while flame and explosion injuries were more frequently recorded in adults, and these differences were statistically significant among patients with documented etiology (χ^2^ = 68.23, *p* < 0.001). This finding should be regarded as hypothesis-generating rather than confirmatory.

The face (70.3%) and upper airway (42.3%) were the most frequently affected anatomical regions. Systematic flexible fiberoptic laryngoscopic evaluation identified mucosal edema in 41.2% of patients. A low intubation rate (4.7%) was observed in this laryngoscopy-evaluated cohort; because all patients underwent laryngoscopy and no non-laryngoscopy comparison group was available, this observation cannot be interpreted as evidence of an association between laryngoscopic evaluation and intubation rate and should not be read as supporting the effectiveness of the laryngoscopic protocol. As discussed in Limitations, this low intervention rate more plausibly reflects the absence of standardized burn-severity data and the heterogeneous, predominantly mild-to-moderate severity spectrum of the cohort. These results are best interpreted as descriptive and hypothesis-generating, underscoring the need for prospective, multicenter studies with standardized documentation of burn severity and etiology to support the development of evidence-based airway management protocols for head and neck burn patients.

## Figures and Tables

**Table 2:** Demographic characteristics of patients with head and neck burns (*n* = 279).

Variable	n	%
Age Groups		
0–2 years	69	24.7
3–6 years	19	6.8
7–12 years	22	7.9
13–17 years	11	3.9
18–40 years	77	27.6
41–65 years	65	23.3
>65 years	16	5.7
Age Category		
Pediatric (<18 years)	121	43.4
Adult (≥18 years)	158	56.6
Sex Distribution		
Male	201	72.0
Female	78	28.0
Seasonal Distribution		
Spring	78	28.0
Autumn	76	27.2
Summer	67	24.0
Winter	58	20.8
Total	279	100.0

Data are presented as numbers (percentages). Seasonal distribution is based on admission date.

**Table 3:** Distribution of burn etiology by age group among patients with documented etiology (analyzable cases, *n* = 162).

Etiology	Pediatric n (%) (n = 68)	Adult n (%) (n = 94)	Total n (%) (n = 162)
Scalding	39 (57.4)	3 (3.2)	42 (25.9)
Electrical	16 (23.5)	22 (23.4)	38 (23.5)
Flame	5 (7.4)	30 (31.9)	35 (21.6)
Explosion	3 (4.4)	22 (23.4)	25 (15.4)
Other *	5 (7.4)	17 (18.1)	22 (13.6)
Total	68 (100)	94 (100)	162 (100)

* Other includes chemical, contact, and hot-oil burns. Cases with undocumented etiology (*n* = 117, 41.9% of the full cohort) were excluded from inferential statistical analysis. Chi-square test: χ^2^ = 68.23, df = 4, *p* < 0.001.

## Data Availability

The data presented in this study are available upon request from the corresponding author, subject to institutional and ethical approval, owing to privacy and ethical restrictions.

## Supplementary Materials

The following supporting information can be downloaded at https://www.mdpi.com/article/10.3390/medicina62101837/s1, Table S1: Aggregate characteristics of patients requiring definitive airway intervention (n = 18). Table S2. Diagnostic performance of mucosal edema and stridor for definitive airway intervention (n = 279).
