# Supplementary material for: Epidemiological and Laryngoscopic Characteristics of Head and Neck Burns Requiring Airway Evaluation: A Single-Center Retrospective Cohort
Source: Medicina (Kaunas). 2026 Sep 23;62(10):1837. doi: 10.3390/medicina62101837 (PMC13707369; doi:10.3390/medicina62101837)
Supplement: Supplementary file 1 [file medicina-62-01837-s001.zip › medicina-4520311-supplementary.pdf]

Table S1. Aggregate characteristics of patients requiring definitive airway intervention (n = 18).

**Characteristic**	**n/N**	**%**
Age category: Adult	14/18	77.8
Age category: Pediatric	4/18	22.2
Etiology: Flame or explosion	12/18	66.7
Etiology: Other/undocumented	6/18	33.3
Facial burn involvement	15/18	83.3
Concurrent upper airway involvement on laryngoscopy	18/18	100.0
Mucosal edema on laryngoscopy	18/18	100.0
Stridor present	7/18	38.9
Intervention type: Endotracheal intubation	13/18	72.2
Intervention type: Tracheostomy	5/18	27.8

Figures are derived from aggregate data already reported in the text and are not a substitute for a patient-level listing.

Table S2. Diagnostic performance of mucosal edema and stridor for definitive airway intervention (n=279)

Finding	Sensitivity (95% CI)	Specificity (95% CI)	PPV (95% CI)	NPV (95% CI)
Mucosal edema	100.0% (82.4–100.0)	62.8% (56.8–68.5)	15.7% (10.1–23.4)	100.0% (97.7–100.0)
Stridor	38.9% (20.3–61.4)	100.0% (98.5–100.0)	100.0% (64.6–100.0)	96.0% (92.9–97.7)
